# Cytotoxicity of Poly(Alkyl Cyanoacrylate) Nanoparticles

**DOI:** 10.3390/ijms18112454

**Published:** 2017-11-18

**Authors:** Einar Sulheim, Tore-Geir Iversen, Vu To Nakstad, Geir Klinkenberg, Håvard Sletta, Ruth Schmid, Anne Rein Hatletveit, Ane Marit Wågbø, Anders Sundan, Tore Skotland, Kirsten Sandvig, Ýrr Mørch

**Affiliations:** 1 SINTEF Materials and Chemistry, Sem Sælands vei 2A, 7034 Trondheim, Norway; vu.nakstad@sintef.no (V.T.N.); geir.klinkenberg@sintef.no (G.K.); havard.sletta@sintef.no (H.S.); ruth.b.schmid@sintef.no (R.S.); annerein.hatletveit@sintef.no (A.R.H.); ane.marit.wagbo@sintef.no (A.M.W.); yrr.morch@sintef.no (Ý.M.); 2 Department of Physics, Norwegian University of Science and Technology, Høgskoleringen 5, 7491 Trondheim, Norway; 3 Department of Molecular Cell Biology, Institute for Cancer Research, Oslo University Hospital—The Norwegian Radium Hospital, 0379 Oslo, Norway; Tore-Geir.Iversen@rr-research.no (T.-G.I.); tore.skotland@rr-research.no (T.S.); kirsten.sandvig@ibv.uio.no (K.S.); 4 Center for Cancer Biomedicine, Faculty of Medicine, University of Oslo, 0379 Oslo, Norway; 5 Department of Cancer Research and Molecular Medicine, Norwegian University of Science and Technology, 8905 MH, 7491 Trondheim, Norway; anders.sundan@ntnu.no; 6 Department of Biosciences, Faculty of Mathematics and Natural Sciences, University of Oslo, 0316 Oslo, Norway

**Keywords:** nanoparticles, PACA, cytotoxicity, nanotoxicology, high-throughput screening

## Abstract

Although nanotoxicology has become a large research field, assessment of cytotoxicity is often reduced to analysis of one cell line only. Cytotoxicity of nanoparticles is complex and should, preferentially, be evaluated in several cell lines with different methods and on multiple nanoparticle batches. Here we report the toxicity of poly(alkyl cyanoacrylate) nanoparticles in 12 different cell lines after synthesizing and analyzing 19 different nanoparticle batches and report that large variations were obtained when using different cell lines or various toxicity assays. Surprisingly, we found that nanoparticles with intermediate degradation rates were less toxic than particles that were degraded faster or more slowly in a cell-free system. The toxicity did not vary significantly with either the three different combinations of polyethylene glycol surfactants or with particle size (range 100–200 nm). No acute pro- or anti-inflammatory activity on cells in whole blood was observed.

## 1. Introduction

Great efforts are being placed on designing nanoparticles (NPs) that can increase the delivery of drugs to cancer cells and facilitate better therapeutic efficacy and more sensitive and specific imaging. Poly(alkyl cyanoacrylate) (PACA), first developed and approved as a surgical glue, was later proven to be a promising drug carrier due to its high loading capacity and biodegradability [1], and is currently being used in multiple late-stage clinical trials [2,3]. PACA NPs are biodegradable, and their degradation rate in cell culture, ranging from a few hours to several days, can be controlled by the choice of production method and the monomer used [4].

Although in vivo toxicity must be assessed during pre-clinical testing of a drug carrier, time and money can be saved if toxic effects are screened early in the research and development process. However, there is a general lack of consensus when it comes to critical characterization parameters needed for determining the toxic effects of NPs [5]. Even though in vitro cytotoxicity cannot be directly correlated to in vivo toxicity, it is generally accepted that in vitro toxicity screening of NP formulations is useful as a rapid and cost-efficient initial evaluation of toxic effects [6,7].

Evaluating NP toxicity is challenging because the toxic effects can originate from both intact NPs and their degradation products. For intact and partly degraded NPs the size, shape, surface charge, surface molecules and core materials can all be the source of toxic effects. Furthermore, the route of cellular entry and intracellular accumulation of the NPs is also expected to be important [8]. As seemingly small changes in NP chemistry can cause dramatic differences in potency and safety, it is of utmost importance to thoroughly characterize NP interactions both in vitro, with cells, and in vivo, following injection in animals [9].

Although there are numerous studies describing the cytotoxicity of PACA NPs [10,11,12], the results generally lack consistency and evaluate only a subset of the PACA NP family. Currently, the literature is lacking a comprehensive and systematic study comparing cytotoxicity of various PACA NPs and the dependency on cell lines. Further, literature describing the underlying mechanisms for cytotoxicity of PACA NPs is also surprisingly scarce.

A comprehensive view of the cytotoxicity of PACA NPs was achieved by synthesizing 19 PACA NPs that varied in terms of composition (*n*-butyl-, 2-ethyl-butyl- or octyl cyanoacrylate (PBCA, PEBCA, and POCA, respectively)), size (100–200 nm), and polyethylene glycol (PEG, three different combinations were used) stabilizers. Their cytotoxic effect on 12 different cell lines was measured utilizing a robotic liquid handling workstation and the CellTiter-Glo^®^ assay to measure the adenosine triphosphate (ATP) level. In a subset of the cell lines and NPs, the toxicity was also evaluated using the 3-(4,5-dimethylthiazol-2-yl)-2,5-diphenyltetrazolium (MTT) and lactate dehydrogenase (LDH) assays. The methods and the cell lines chosen are frequently used in research and/or are part of the assay cascade developed and used by the Nanotechnology Characterization Laboratory at the National Cancer Institute (NCI-NCL) in the US and adapted by the European Nanomedicine Characterization Laboratory (EU-NCL).

## 2. Results

### 2.1. Physico-Chemical Properties of PACA NPs

The physico-chemical properties of the particles produced are summarized in Table 1. All particles had a hydrodynamic diameter (in 0.01 M phosphate buffer, pH 7.0) between 100 and 200 nm with a polydispersity index (PDI) below 0.26. The particles did not aggregate in either PBS or the cell culture medium. The size of the NPs produced was found to depend somewhat on the composition, increasing with the molecular weight (MW) of the monomers (BCA < EBCA < OCA) and only slightly increasing with the MW of the PEG. The NPs of different monomers could, therefore, not be distributed equally into different-sized groups. The surface charge of PEGylated NPs was slightly negative, ranging from −7 to −1 mV, indicating dense PEGylation [13].

The molar mass distribution of polymer chains in the various NPs was determined by size exclusion chromatography (SEC). The average molecular weight (M_n_) was found to be similar (48,000–53,000 g/mol) for the three different polymer materials used in the study (PBCA, PEBCA and POCA). Calculating average chain length from M_n_ showed that PBCA NPs were comprised of slightly longer polymer chains than PEBCA and POCA NPs (Table 1).

### 2.2. High-Throughput Cytotoxicity Screening

As toxicity can be very cell line-dependent we performed high-throughput cytotoxicity screening of our PACA NPs in the 12 cell lines listed in Table 2.

**Figure 1:**
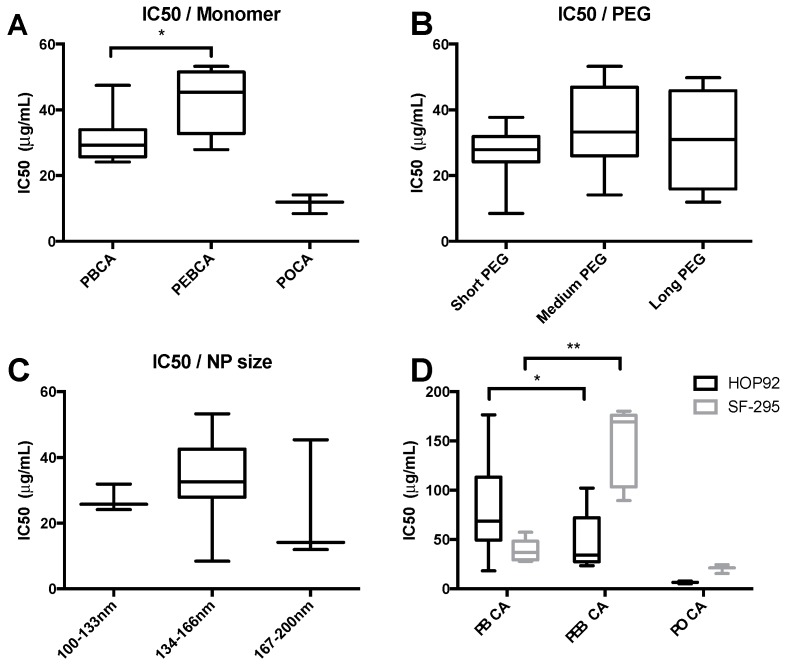
Box plots of IC_50_ values from the HTS setup using CellTiter-Glo^®^ on a large number of cell lines (given in Table 2). Incubation time was 24 h. (**A**–**C**) IC_50_ values over all 12 cell lines with NPs grouped by material (**A**); Pegylation ((**B**); short, medium, and long PEG is Kolliphor/Brij, Jeffamin/Brij, and Kolliphor/Pluronic, respectively); and size (**C**); (**D**) example of differing tolerance to different monomers in two cell lines. * *p* < 0.05, ** *p* < 0.005. POCA NPs are significantly different from the other NPs in both cell lines in (**A**,**D**). Nineteen different NPs are included, the size of the various groups is found in Table 2. Central line shows median value, boxes show 1^st^ and 3^rd^ quartiles and whiskers shows min and max values.

All 19 NPs were screened for cytotoxic effects after 24 h incubation at concentrations ranging from 0.3 to 285 μg/mL using an HTS setup and detecting ATP content using the CellTiter-Glo^®^ assay. The resulting mean IC_50_ values listed in Table 2 demonstrate large variations between the different cell lines. In order to obtain a better understanding of the varying toxicity, the NPs were grouped based on their composition (monomer), surface functionalization (PEGylation), and size. Toxicity values from the grouped variables are given in Figure 1A–C. Interestingly, no significant differences were found for the three different combinations of PEGylation or for testing NPs with different sizes in the range 100–200 nm. However, the particle composition, i.e., the type of monomer used, was found to affect the cytotoxicity, with PEBCA NPs being the least toxic, followed by PBCA and POCA NPs with mean IC_50_ values ranging from 68 μg/mL for PEBCA to 12 μg/mL for POCA NPs. It is noteworthy that some cell lines had the highest tolerance towards PBCA NPs, e.g., HOP92 (Figure 1D). Large differences in cytotoxicity were observed for the various types of NPs (i.e., different materials) between the various cell lines. This variation was especially noticeable for PEBCA NPs where the IC_50_ values spanned from 18 μg/mL in ovarian carcinoma cells (OVCAR-3) to above 300 μg/mL for prostate carcinoma cells (DU-145). Notably, in the latter cell line the tolerance of PEBCA NPs was more than 10× higher than for both PBCA and POCA NPs.

**Figure 2:**
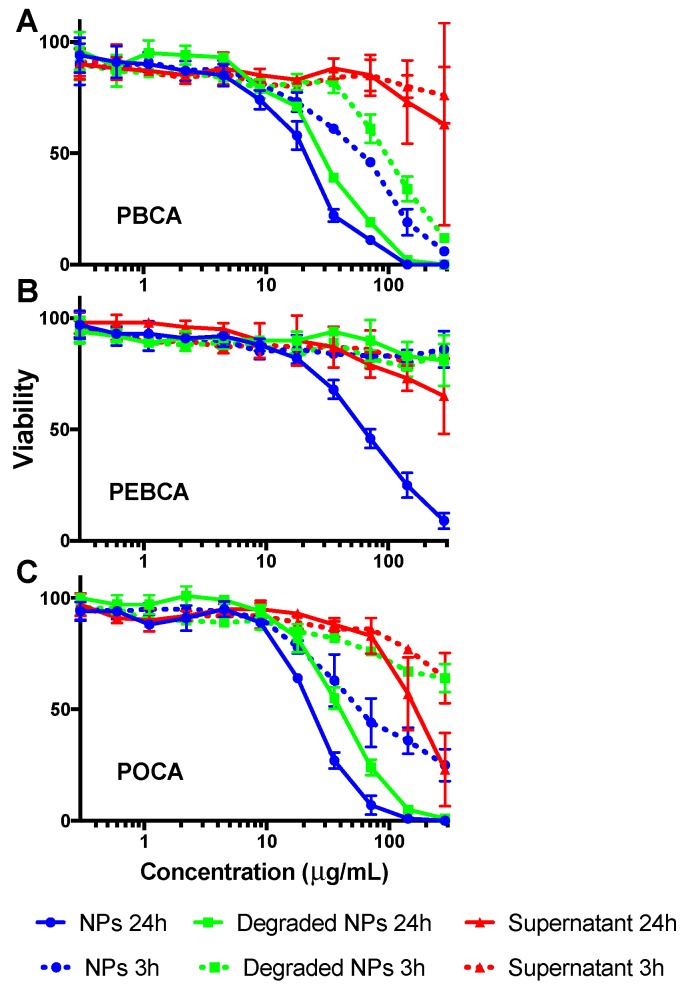
Toxicity of intact NPs (blue), degraded NPs (green), and supernatant from centrifuged and partly degraded NPs (red) after 3 h (dotted line) and 24 h (continuous line) in Hep G2 cells measured using the CellTiter-Glo^®^ assay. (**A**–**C**) show results from PBCA, PEBCA, and POCA NPs, respectively. Each point is the average from two different NPs with the same monomer, but with different PEGylations (short and long PEG, respectively). Error bars show the standard deviation.

Previously, the toxicity of PACA NPs has been attributed to the degradation products originating from bioerosion [12]. NPs and NP degradation products removed from circulation are mosty found in the liver. Hence, the toxicity of both NP components and NP degradation products was evaluated by incubating Hep G2 cells for both 3 h and 24 h with (i) intact NPs; (ii) degraded NPs; and (iii) the supernatant obtained after centrifugation of NPs pre-incubated in cell culture medium for 24 h (Figure 2). These analyses revealed that (i) the intact NPs were most cytotoxic; (ii) the supernatant was only toxic at very high concentrations; and (iii) the degraded NPs were less toxic than intact NPs, especially for PEBCA. The toxicity of the PEG-based surfactants was also measured giving some toxicity around 10 μg/mL (Figure S1), which is 10–100 times higher than the expected concentration of surfactants in the NP suspensions. In Hep G2, as in most cell lines, PEBCA was found to be the least toxic of the three materials tested.

**Figure 3:**
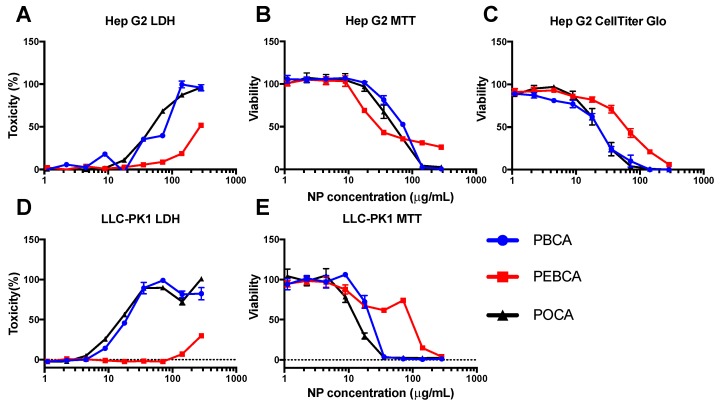
LDH (**A**,**D**); MTT (**B**,**E**) and CellTiter-Glo^®^ (**C**) tox assay in Hep G2 (**A**–**C**) and LLC-PK1 (**D**,**E**) cells after 24 h of exposure to PACA NPs. Error bars show the standard deviations (*n* = 4).

While the full screen was performed using CellTiter-Glo^®^, an assay based on ATP measurements, cytotoxicity was also evaluated using the MTT and LDH assays in Hep G2 and LLC-PK1 cells as these methods are part of the standardized test regime used by NCI-NCL for toxicity profiling of nanomaterials [14]. The MTT assay provides an estimate of the metabolic activity of the cell by measuring the reduction of MTT, while LDH analysis is an assay for the quantification of cell lysis by measuring release of LDH from the cytosol of damaged cells. Figure 3A,D show that the results for the LDH-analysis were similar to that obtained with CellTiter-Glo^®^ (Figure 3C), namely that PBCA and POCA NPs are more toxic than PEBCA NPs. This might indicate that the toxicity seen for PBCA and POCA NPs acts through damage to the cell membrane. In addition, the concentration at which various toxicity levels were found with LDH measurements (e.g., IC_50_) was very similar to that obtained with CellTiter-Glo^®^. In contrast, using the MTT assay (Figure 3B,E), PEBCA NPs were found to be more toxic than the two other NPs in Hep G2 cells supporting that other cellular mechanisms are involved in the cell’s reaction to PEBCA NPs than to PBCA and POCA NPs. No interaction between the NPs and the assay was observed for any of the three assays.

### 2.3. Is Toxicity Due to Cellular Association/Uptake of NPs?

**Figure 4:**
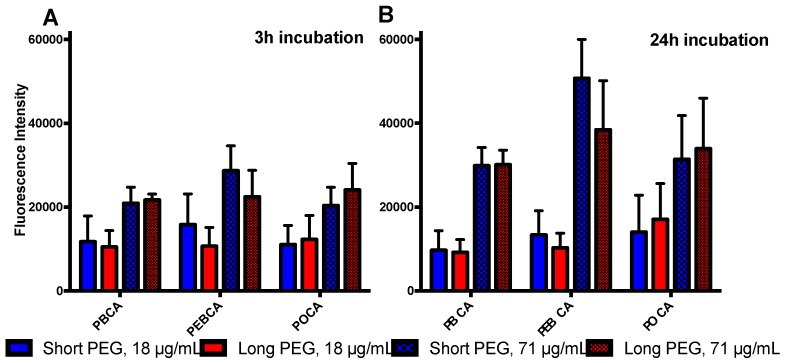
Cellular association of NPs averaged over the three cell lines Hep G2, IMR-90 and HeLa dynK44A. Nontoxic concentration: 18 μg/mL; toxic concentration: 71 μg/mL. (**A**) 3 h incubation; (**B**) 24 h incubation. Error bars show the standard deviation.

**Figure 5:**
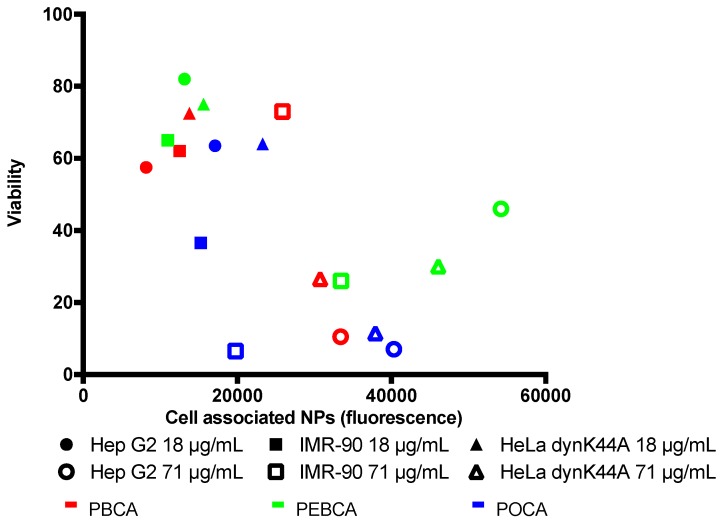
Cellular association of NPs plotted against viability of three different cell lines, Hep G2, IMR-90, and HeLa dynK44A, incubated with NPs of PBCA (red), PEBCA (green) and POCA (blue) for 24 h, measured using the CellTiter-Glo^®^ assay. Each point is the average of two different NPs of the same monomer, but with different PEGylations (long and short PEG); 18 μg/mL is considered non-toxic, while 71 μg/mL is a toxic concentration. HeLa dynK44A had tetracycline in the cell medium and, hence, were functioning normally.

Since intact NPs were found to be the primary reason for the observed cytotoxicity, we tested if this was due to cellular uptake/association in three cell lines, Hep G2, IMR-90, and for the HeLa dynK44A, both with and without tetracycline. The HeLa dynK44A cell line is stably transfected with a plasmid encoding a dominant-negative mutant of dynamin, DynK44A. Inhibition of dynamin-dependent endocytosis by overexpression of mutant dynamin is regulated by a tetracycline-binding transactivator that binds to the promoter region in the absence of tetracycline. Thus, in cells grown with tetracycline only endogenous dynamin is expressed, whereas in the absence of tetracycline the cells overexpress the dominant interfering dynamin mutant and dynamin-dependent endocytosis will be inhibited [15]. Figure 4 shows the association of different fluorescently-labeled NPs given by the fluorescence intensity at two different particle concentrations (non-toxic 18 μg/mL and toxic 71 μg/mL) and after 3 h and 24 h of incubation. Interestingly, for the non-toxic concentration, the main increase in cellular uptake/association was observed within the first 3 h of incubation. In contrast, at toxic concentrations, there was a significant increase in fluorescence from 3 to 24 h. No significant differences were found between NPs with different PEGylation (short, medium, or long). However, the degree of association was found to be dependent on the polymer material, but only at toxic concentrations. At 71 μg/mL (toxic concentration) there was a clear trend that higher amounts of PEBCA NPs were found associated with cells compared to PBCA and POCA NPs. It is to be noted that significantly higher IC_50_ values were obtained for the PEBCA NPs and at this concentration (and depending on the NP toxicity), many of the cells were deformed or detached from the plate and, hence, gated out. Indeed, much fewer cells were found in the viable population. Therefore, this result does not indicate a more rapid uptake or association of PEBCA NPs, but rather that the cells can remain viable with a higher load of PEBCA NPs compared to PBCA and POCA NPs. At the non-toxic concentration, no difference in association/uptake by the cells was observed. By comparing cell-associated fluorescence to viability (Figure 5), it was observed that high fluorescence generally corresponded to lower viability. The cell associated fluorescence and toxicity after incubation at 4 °C was also assessed, but resulted in very low fluorescence and no toxicity after 3 h, indicating that energy-dependent processes are involved in the association/uptake (data not shown).

**Figure 6:**
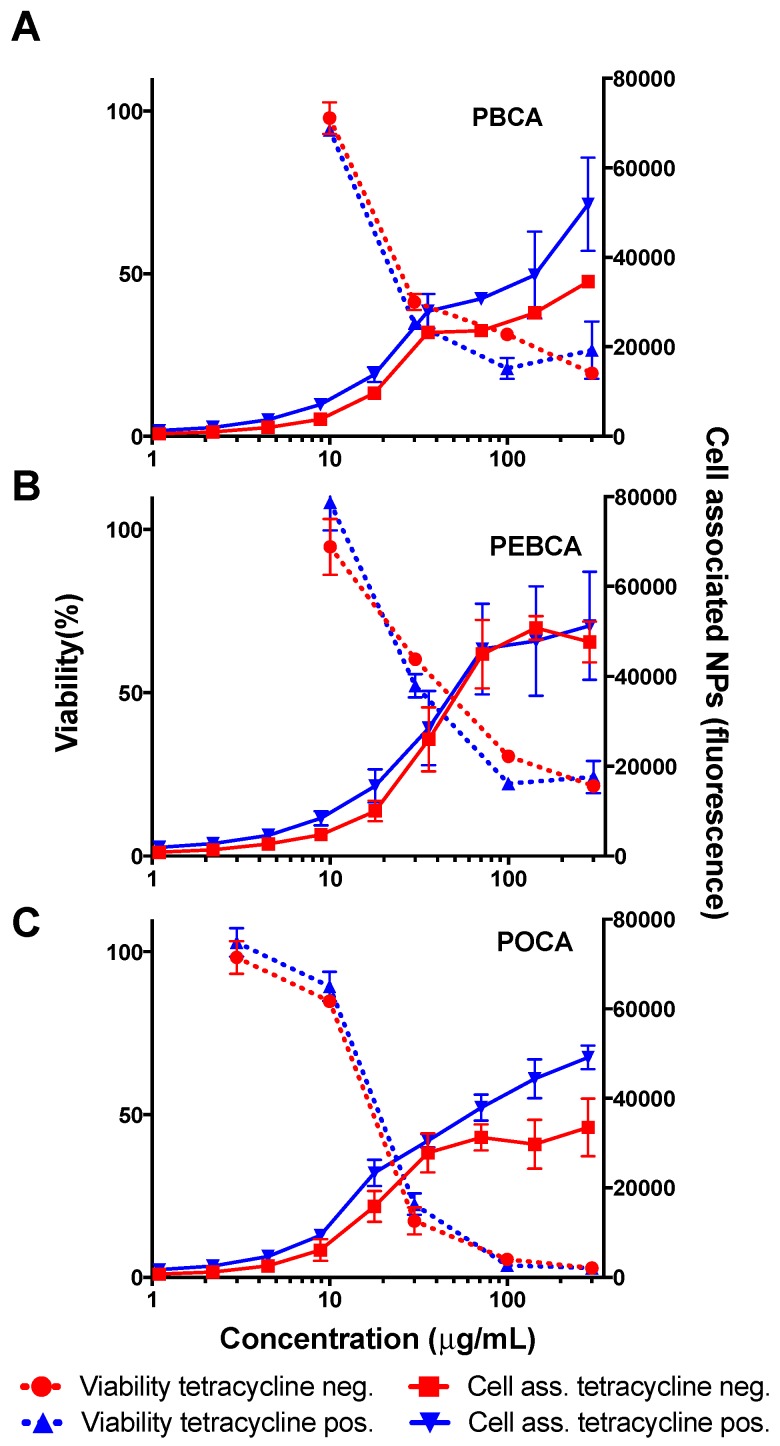
Cellular association/uptake (right axis, continuous line) and related toxicity (left axis, dotted line) of PBCA (**A**), PEBCA (**B**) and POCA (**C**) NPs in HeLa dynK44A cells with (blue lines) and without (red lines) tetracyclin (1 μg/mL) after 24 h incubation. Incubation without tetracycline inhibits dynamin-dependent endocytosis. Association/uptake is averaged from two NPs of the same material while viability is averaged from three experiments with the standard deviation shown.

To further evaluate the link between cellular uptake/association and toxicity the HeLa dynK44A cell line was incubated with the three different NPs in presence or absence of tetracycline added to the medium. As shown in Figure 6, the inhibition of dynamin-dependent endocytosis only gave a limited reduction in cell-associated fluorescence, and what seems to be a modest reduction in toxicity. Thus, the results do not show a clear correlation between reduced dynamin-mediated cellular uptake/association and viability. It should be noted that due to the low number of viable cells at higher NP concentrations the measured cellular association above 30–40 μg/mL is uncertain.

**Figure 7:**
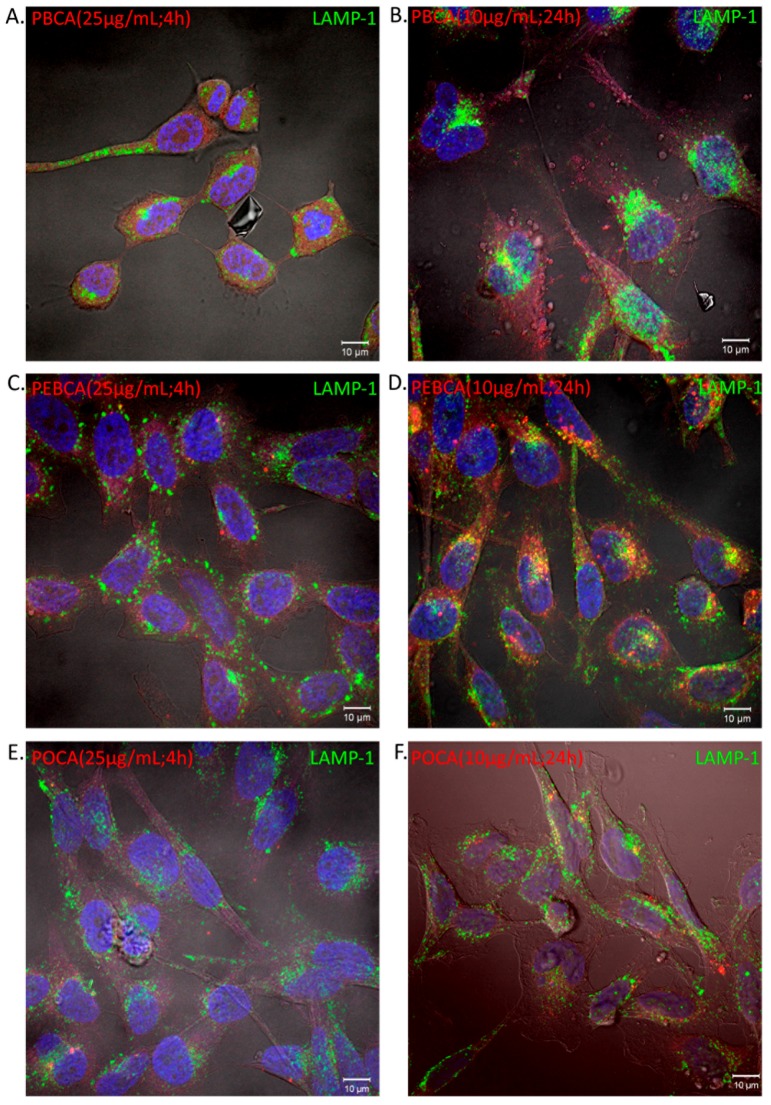
Binding and uptake of the PACA NPs to cells. (**A**) PBCA 25 μg/mL; (**B**) PBCA 10 μg/mL; (**C**) PEBCA 25 μg/mL; (**D**) PEBCA 10 μg/mL; (**E**) POCA 25 μg/mL; (**F**) POCA 10 μg/mL. The HeLa cells were incubated with the PACA NPs (PBCA, PEBCA, and POCA labelled with dye NR668, in red) at 37 °C for 4 h and 24 h. The cells were fixed (paraformaldehyde) and stained with an antibody against LAMP-1 followed by the appropriate secondary ab-alexa488 (green) conjugate. The cell nuclei were stained with Hoechst 33342 (in blue). Co-localization of NPs and lysosomes are indicated by yellow areas.

Confocal microscopy was performed to investigate whether the observed NP fluorescence originated from internalized or membrane-associated NPs. HeLa cells were incubated with sub-toxic concentrations (10 and 25 μg/mL) of the three different NR668-stained PACAs for 4 and 24 h. The fluorescence is mainly observed from inside the cell showing that the NPs are indeed internalized and not surface associated. At the highest concentration, most of the cells incubated with PBCA NPs changed to a ‘rounded’ morphology already within 2 h, and at these early time points (2–4 h) the cells displayed a very faint NR668 staining with no significant uptake (vesicular staining) or co-localization with the lysosomal marker LAMP-1 (Figure 7).

Furthermore, a change in the cellular localization of lysosomes occurred depending on which NPs the cells were exposed to. In the cells incubated with PBCA NPs, a clustering of lysosomes into larger structures compared to control cells seemed to occur when cells were incubated with sub-toxic NP concentrations. This effect could also be seen for PEBCA NPs at 25 μg/mL. In the cells exposed to PBCA NPs, lysosomes displayed condensed and polarized perinuclear localization, whereas in the cells exposed to POCAs and PEBCAs, NPs’ lysosomes had a more evenly distributed localization similar to that observed in control cells without NPs. These effects on the lysosomes occurred quite rapidly (<4 h), when little or no significant uptake of the PACA NPs was observed. After incubation of cells with the PACA NPs for 24 h, cells with PEBCA NPs (10 μg/mL) displayed only a slightly changed morphology and significant PEBCA internalization was observed both as a vesicular staining and with LAMP-1 co-localization (Figure 7). The PBCA and POCA NPs displayed hardly any vesicular staining and an insignificant LAMP-1 co-localization.

### 2.4. Sterility and Immunology

Various inflammation assays were used to examine PEBCA NP's ability to generate acute inflammation responses (see Materials and Methods in Supplementary Material S2). No acute pro-inflammatory effect was observed on isolated human monocytes (by measuring the TNF-α secretion) when incubated with PEBCA NPs (2–20 μg/mL). Human whole blood assay was used to measure CD11b in granulocytes and monocytes and the stimulation of cytokine production. No acute pro- or anti-inflammatory activity on cells in whole blood was observed at the NP concentrations tested (up to 50 μg/mL, Supplementary Material S2). The endotoxin content in the particle samples was found to be negligible, ranging from 0 to 17 pg/mL (0–0.17 EU/mL) at particle concentrations ranging from 2 to 200 μg/mL.

## 3. Discussion

Although there are several thorough studies on PACA toxicity [11,16], the extent of these studies is often limited to one or two cell lines and few NP variations. Additionally, for some alkyl cyanoacrylate monomers, especially isohexyl cyanoacrylate, multiple chemical structures are possible, making the existing literature difficult to interpret. By using a high throughput screening methodology, we wanted to assess the toxicity of different NP batches on various cell lines to help in both selecting the least toxic NP for further work, and to investigate the variation between cytotoxicity assays. We produced an array of nanoparticles covering a range of properties and selected cell lines frequently used in the literature. Cytotoxicity has been shown to be very cell-line dependent [10,17]. This might be due to differences between cell lines in their ability to interact and endocytose the nanoparticles, and in the fact that cancer cell lines in particular often have deregulated pathways of cell signaling, cell death (apoptosis), and autophagy [18,19].

### 3.1. NP Toxicity

Average IC_50_ for the three types of PACA NPs (PBCA, PEBCA, POCA) was found to be 31, 68, and 12 μg/mL respectively. This is slightly higher than reported by Lira et al., [16] and significantly lower compared to the data from Kolter et al. [11]. As previously reported for PACA, the monomer has significant impact on the toxicity of otherwise similar NPs [12]. This was attributed to differences in the degradation rate explained by rapidly-degrading NPs resulting in a high local concentration of released polymer chains. Hence, this has been the accepted explanation to the generally observed higher toxicity of PBCA compared to PEBCA and POCA. However, we report here that POCA, the slowest degrading polymer of the three materials used in the current study [4], also causes the highest cytotoxic effect. PEBCA, with an intermediate degradation, shows the lowest toxicity. This non-linearity in the toxicity of PACA NPs with increasing organic backbone is surprising and is, to our knowledge, not previously reported. It should be noted that the POCA NPs used in this study were prepared from three different batches of OCA to avoid artifacts from contaminations of the chemicals as pointed out as a common pitfall in NP characterization [20].

Interestingly, cytotoxicity of the completely degraded NPs did not correlate with that of the corresponding intact NPs. The degraded PEBCA NPs showed no significant toxicity, whereas degraded PBCA and POCA NPs were 1.5–2-fold less toxic than their intact NP counterpart. This clearly demonstrates that the toxicity, even for rapidly degrading NPs, cannot be assessed by its constituents alone, and illustrates the importance of testing toxicity both of intact NPs, as well as partly or fully degraded particles. The difference in toxicity observed for the NPs cannot be attributed to varying polymer chain length as these were within the same range (Table 1).

While the toxicity of PBCA compared to PEBCA might be explained by faster degradation and a more rapid release of degradation products, this cannot explain why POCA, previously shown to be stable for at least six days in cells and cell medium [4], gives higher cytotoxicity. To understand this, one needs to investigate how cells handle internalization of slowly-degrading NPs. Detailed mechanistic studies are beyond the scope of this study, but we have indications showing that formation of intracellular aggregates and upregulation of heat shock proteins might be involved (unpublished results). It has been previously shown that degradability is important for the toxic effect of NPs following internalization [21]. The different alcohols produced during degradation (butanol, isohexanol, and octanol) could contribute to the differences in toxicity; although it has been shown previously [12] that butanol from degrading PBCA particles has no acute cytotoxic effect. Furthermore, it cannot be ruled out that the length of the organic backbone, and hence its lipophilicity, might play a role in cytotoxicity (with OCA being more lipophilic than EBCA).

Within the range of NP size- and surface charge evaluated here (100–200 nm, −7 to −1 mV) these parameters had no significant effect on the measured toxicity. Size-dependent toxicity is frequently reported for inorganic NPs [22,23], and also documented for PACA NPs by Lherm et al., [12] who showed that 50 nm NPs were more toxic than 200 nm NPs. Our study was limited to NPs in the range of 100–200 nm which might explain why the same size-dependent toxicity was not observed in our study. Interestingly, we found that the type of PEG used did not affect the toxicity, and the PEG molecules alone were found toxic only at concentrations 10–100 times higher than found in the NP dispersions. However, this does not necessarily mean that the PEG molecules cannot affect the cells differently when presented by and released from a NP [24], and we have previously shown that the PEG affects the cellular uptake of these NPs [25].

### 3.2. The Role of Cellular Uptake/Association on Toxicity

In the current study flow cytometry was used to measure the cellular association of NPs at various time points and concentrations. Although a single-cell technique, this cannot be used to separate internalized NPs from those that are membrane-bound, and there is the possibility that only the dye is transferred to the cell through contact-mediated transfer [26,27]. However, the absence of fluorescence in cells after incubation at 4 °C suggests that the fluorescence remains in the nanoparticle [26], and confocal microscopy showed that the observed fluorescence is mainly due to internalized NPs taken up through energy-dependent processes as also previously observed [4].

Our studies do not indicate that lower uptake in cells is the reason for the apparent lower toxicity of PEBCA NPs. On the contrary, at toxic NP concentrations we observed far higher fluorescence from the cells incubated with PEBCA NPs. This is likely due to the ATP depletion in apoptotic cells inhibiting the energy-dependent uptake of POCA and PBCA NPs. In addition, RBE4, a cell line previously shown to have a very high uptake of these NPs [3], is above average in tolerance to the NPs (Table 2). In HeLa dynK44, inhibiting dynamin-dependent uptake gave a weak indication of a connection between uptake and toxicity as the small reduction in uptake was found to coincide with a modest increase in viability, but these results are indecisive as the reduction in uptake was very limited. However, confocal images of HeLa cells incubated with the various NPs suggested that the cells were affected by cell associated NPs as both the intracellular distribution of lysosomes and the cell morphology changed with increasing NP concentration. This was especially prominent for PBCA NPs and might be closely related to the low ATP levels measured after incubation with these NPs. Lysosomal clustering and perinuclear localization, which was mainly observed for PBCA NPs, has been shown to occur in starving cells [28].

### 3.3. Evaluating In Vitro Toxicity

Understanding the toxicity of NPs is challenging, and our studies show that no single property can explain the observed toxic effects of PACA NPs. There are indications that the cells are challenged both by rapid release of polymers, and by internalized, slowly degrading NPs. Surprisingly, PEBCA, with the intermediate degradation rate of the three PACA NPs studied, was found to be the least toxic of these NPs.

Reliable methods for safety assessment of nanomaterials are critical both for those intended for medical use and drug encapsulation, but also for NPs used in other products. The latter has gained significant focus and the need for robust screening methods have been highlighted in multiple reviews [29,30]. In this study, we have seen that not only the cell line, but also the method used for measuring cytotoxicity, largely affects the outcome of the result. In a subset of the cells, and following the NCI-NCL standard, toxicity was also measured using MTT and LHD. In both cell lines it was seen that PEBCA, the least toxic NP gave much less membrane damage (through the LDH assay) compared to PBCA and POCA. MTT, on the other hand, indicated toxic effects also from PEBCA below the concentrations found using LDH or CellTiter-Glo^®^. This highlights the complexity of nanotoxicology and clearly shows that cytotoxicity of NPs depends both on the toxicity assay used and the cell line tested.

## 4. Methods

### 4.1. Nanoparticle Synthesis

All NPs were prepared using the mini-emulsion polymerization method as described previously [31] and a detailed protocol for the synthesis and chemicals used is found in the Supplementary Materials. Briefly, NPs of PBCA, PEBCA, or POCA were made by mixing the oil phase, consisting of the monomer, a neutral oil, and fluorescent dye (NR668 [32] or pentamer hydrogen thiophene acetic acid methyl ester (pHTAM) [33]), with the aqueous phase consisting of hydrochloric acid (HCl) and the PEG-surfactants. Three different combinations of PEG surfactants were used, Kolliphor^®^ HS15 and Brij^®^ L23 (short PEG), Jeffamine^®^ M-2070 and Brij^®^ L23 (medium PEG), and Kolliphor^®^ HS15 and Pluronic^®^ F68 (long PEG) [13]. The oil in water mini-emulsion was made using a tip sonifier. The polymerization was then carried out overnight and unreacted monomer and surplus of surfactants were rinsed by extensive dialysis.

The size, size distribution and ζ-potential were determined using dynamic and electrophoretic light scattering (Zetasizer Nano ZS, Malvern Instruments, Malvern, UK) in 0.01 M phosphate buffer, pH 7. The reported NP size is the *Z*-average.

### 4.2. NP Pre-Preparation for Toxicity Measurements

We evaluated the toxicity of intact NPs, degraded NPs, the NP diluent, and of the surfactants alone. The different PEG-surfactants were dissolved directly in the cell culture medium and added to the cells. Toxicity of the surfactants was measured in HeLa cells after 24 h incubation using the CellTiter-Glo^®^ assay (Promega, Madison, WI, USA). For assessment of NP degradation products, the NPs were diluted in glycine buffer (pH 9.0, 0.2 M) and placed at 50 °C for several days to achieve total NP degradation, this is referred to as degraded NPs. To assess the toxicity of material released from partly degraded NPs and the NP diluent, particles were also placed in cell culture medium at 37 °C for 24 h followed by ultracentrifugation and collection of the supernatant.

### 4.3. Cell Culture Conditions

The cells were cultivated at 37 °C and kept in the log-phase during the experiments. Growth media for the cell lines used in high-throughput cytotoxicity screening are shown in Table S1.

### 4.4. High Throughput Screening Assays

For high-throughput cytotoxicity measurements, cells were seeded in 384-well plates (Corning, New York, NY, USA) at densities shown in Table S1, and cultivated for 24 h before addition of NPs. After 3 or 24 h of incubation with NPs ranging in concentration from 0.3 to 285 μg/mL, CellTiter-Glo^®^ (20 μL) was added and incubated with the cells for 10 min before the luminescence from each well was recorded using a spectrophotometer (Tecan, Zurich, Switzerland). Cell medium alone was used as the negative control and a serial dilution of digitonin ranging from 0.002 to 140 μg/mL was used as the positive control. Degraded NPs and the supernatants obtained after centrifugation of the nanoparticles at 25,000 rpm for 120 min (Sorvall WX Ultra 80, Thermo, Waltham, MA, USA) were tested in Hep G2 cells at volumes corresponding to those used for NPs. Each reported value is the average from 4 wells. All steps were performed by a high-throughput screening (HTS) system (Tecan Freedom EVO, Zurich, Switzerland), in-house modified to minimize differences due to pipetting/handling.

In order to test the observed toxicity in standardized setups, the nanoparticles were analyzed with MTT and LDH following the NCI-NCL GTA 001 and 002 standards [14]. Here, Hep G2 and LLC-PK1 cells were seeded in 96-well plates and incubated for 24 h to 80% confluency. NPs at nine different concentrations (1–285 μg/mL) were then added to the wells (three parallels) and incubated for 24 h. Triton X-100 was added as a positive control and the plate was incubated for an additional 10 min before 50 μL of the cell medium were removed and used for analysis of LDH as described below. The rest of the cell medium was exchanged with fresh medium before 50 μL of MTT (3-(4,5-dimethyl-2-thiazolyl)-2,5-diphenyl-2*H*-tetrazolium bromide, Sigma, St.Louis, MO, USA) were added. After 4 h the liquid was exchanged with 200 μL DMSO and 25 μL of glycine buffer (pH 10.5, 0.1 M) and the absorbance at 570 nm was measured using a plate reader.

For analysis of LDH, 50 μL of the prepared LDH solution (Biovision LDH-cytotoxicity assay kit, Biovision, Milpitas, CA, USA) were added to 50 μL of the cell medium and incubated for 20 min before the absorbance at 490 nm was measured using a plate reader.

### 4.5. Uptake Studies by Flow Cytometry

Cellular uptake/association was measured in Hep G2, HeLa DynK44a, and IMR-90 cells by seeding the cells in 96-well plates (Corning). NPs (1–285 μg/mL) were added to the wells 24–48 h after seeding, and incubated for 1–24 h. Cells were incubated at 37 °C, except for the control of energy-dependent uptake/association and dye leakage, where cells were incubated at 4 °C for 3 h. Prior to analysis, the cells were washed twice in PBS. The cells were then trypsinized and kept at 4 °C until analysis. NPs associated with the cells were measured using a flow cytometer (Life Technologies Attune acoustic focusing flow cytometer, Thermo, Waltham, MA, USA) drawing cells directly from the 96-well plates. At non-toxic concentrations, 5000 cells from each well were analyzed. As toxicity increased, the number of cells inside the gate of the viable population decreased. The NPs were labeled with p-HTAM, excited using a 405 laser and detected from 430 to 470 nm. To gate out aggregates and cell debris from the results, an ROI was drawn around the viable population in a side scatter vs. forward scatter plot. In order to compare the cellular uptake of different NPs, the fluorescence intensity from the different NP batches was measured using a spectrophotometer (Biotek Instruments, Winooski, VT, USA) and it was corrected for the differences by multiplying the result with a scaling factor. The NPs had relatively similar fluorescence with scaling factors ranging from 0.95 to 1.35.

### 4.6. Cellular Uptake of PACA NPs by Confocal Fluorescence Microscopy

To further assess cellular uptake and association, HeLa cells (seeded 2 × 10^4^ cells/well in 24-well trays) were cultured on coverslips in full medium for 24 or 48 h prior to incubation of the PACA NPs (10 or 25 μg/mL) loaded with NR668 for 24 or 4 h, respectively. Cells were then fixed in 4% *w*/*v* paraformaldehyde for 15 min and washed twice in PBS containing 0.05% *w*/*v* saponin and 0.2% *w*/*v* BSA. Further, the cells were immuno-stained using the indicated primary antibody (Rabbit anti-LAMP1 (diluted 1:200) from Sigma-Aldrich (L1418)) for 1 h, washed three times in PBS/saponin, stained with the secondary antibody (donkey anti-rabbit-alexa488 (diluted 1:200) conjugate from Jacksons ImmunoResearch Laboratories) for 1 h, and washed three times with PBS. The cells were mounted in Prolong Gold with DAPI (Life Technologies). The fixed cells were examined with a Zeiss LSM710 or LSM780 confocal microscope (CarlZeiss MicroImaging GmbH, Jena, Germany) equipped with an multiline Ar laser (458/488/514 nm), a DPSS-561 10 (561 nm), a 405-30CW laser diode (405 nm), and a HeNe-laser (633 nm). The objective used was a Zeiss plan-Apochromat 63x/1.4 Oil DIC III. Image processing and visualization were performed with ZEN 2010 (Carl Zeiss MicroImaging GmbH) and Photoshop CS4 (Adobe, San Jose, CA, USA).

## 5. Conclusions

In this study we evaluated the cytotoxicity of 19 different PACA NPs, which varied in terms of size, PEGylation and composition/monomer, in 12 different cell lines. We found that PEBCA NPs, with an intermediate degradation rate, was significantly less toxic than both PBCA and POCA NPs (fast and slow degradation rate), and that neither size nor PEGylation affected the toxicity significantly. Interestingly, the observed toxicity originated from intact NPs, not degradation products, but the toxicity could not be explained by differences in cellular uptake or association. We also show that within a range of relatively similar NPs significant variations in reported toxicity can be expected when testing in different cell lines, highlighting the importance of high throughput methodologies to draw reliable conclusions.

## Figures and Tables

**Table 1:** The different groups of nanoparticles (NPs) included in the study. Size groups contain all three polyethylene glycol (PEG)-groups. For molecular weight calculations numbers are given as mean ± standard deviation from *n* = 2. The particle size is the hydrodynamic diameter (*Z*-average). The size and zeta potential was measured at pH 7.0.

Particle	PEG Surfactants	PEG Description	Size Groups	Zeta Potential	Polymer Chain Length (Mn and Monomer Units)
PBCA (*N* = 11)	Kolliphor/Brij (*N* = 4)	Short PEG	100–133 nm (*N* = 3)	(-1) – (-7) mV	50.5 ± 13.2 kD N: 327 ± 86
	Jeffamin/Brij (*N* = 5)	Medium PEG			
			134–166 nm (*N* = 8)		
	Pluronic/Kolliphor (*N* = 2)	Long PEG			
PEBCA (*N* = 5)	Kolliphor/Brij (*N* = 2)	Short PEG	134–166 nm(*N* = 4)	(-1) – (-7) mV	48.0 ± 3.6 kD N: 265 ± 20
	Jeffamin/Brij (*N* = 2)	Medium PEG			
			167–200 nm (*N* = 1)		
	Pluronic/Kolliphor (*N* = 1)	Long PEG			
POCA (*N* = 3)	Kolliphor/Brij (*N* = 1)	Short PEG	134–166 nm (*N* = 1)	(-1) – (-7) mV	53.0 ± 2.3 kD N: 254 ± 11
	Jeffamin/Brij (*N* = 1)	Medium PEG			
			167–200 nm (*N* = 2)		
	Pluronic/Kolliphor (*N* = 1)	Long PEG			

**Table 2:** The 12 cell lines used for high-throughput cytotoxicity screening. Measured tolerances (IC_50_ values; μg/mL) to PACA NPs are given as mean value ± standard deviation. The three first cell lines listed were screened only against a subset of NPs. The average IC_50_ value for prostaste carcinoma cells (DU-145 cells) could not be calculated due to values out of range (>300 μg/mL).

Cell Lines	Cell Origin	IC_50_ All NPs (μg/mL), *N* = 18	IC_50_ PBCA NPs (μg/mL), *N* = 10	IC_50_ PEBCA NPs (μg/mL), *N* = 5	IC_50_ POCA NPs (μg/mL), *N* = 3
IMR-90	Lung fibroblast (normal)	26 ± 19	32 ±2 4	22 ± 7	13 ± 3
Hep G2	Liver carcinoma	26 ± 24	21 ± 3	70 ± 4	24 ± 1
RBE4	Rat brain endothelial cell	44 ± 29	50 ± 5	74 ± 15	6 ± 1
DU-145	Prostate carcinoma	-	30 ± 7	>300	22 ± 8
A498	Kidney carcinoma	23 ± 8	23 ± 5	30 ± 8	12 ± 3
SW-620	Colon adenocarcinoma	37 ± 19	35 ± 7	56 ± 20	10 ± 3
HOP-92	Non-small lung carcinoma	53 ± 45	77 ± 46	31 ± 7	7 ± 1
MDA-MB-468	Mammary gland adenocarcinoma	19 ± 11	20 ± 9	26 ± 10	5 ± 1
SF-295	Glioblastoma multiform	65 ± 58	38 ± 9	145 ± 40	20 ± 5
UACC-62	Mammary gland carcinoma	15 ± 6	16 ± 2	19 ± 8	6 ± 1
OVCAR-3	Ovarian adenocarcinoma	15 ± 7	15 ± 6	18 ± 7	8 ± 1
COLO 205	Colon adenocarcinoma	23 ± 7	22 ± 6	31 ± 3	13 ± 3

## Supplementary Materials

Supplementary materials can be found at www.mdpi.com/1422-0067/18/11/2454/s1.

## References

[1] Vauthier C., Dubernet C., Fattal E., Pinto-Alphandary H., Couvreur P. (2003). Poly(alkylcyanoacrylates) as biodegradable materials for biomedical applications. Adv. Drug Deliv. Rev..

[2] Kumari A., Yadav S.K., Yadav S.C. (2010). Biodegradable polymeric nanoparticles based drug delivery systems. Colloids Surf. B Biointerfaces.

[3] Torchilin V.P. (2014). Multifunctional, stimuli-sensitive nanoparticulate systems for drug delivery. Nat. Rev. Drug Discov..

[4] Sulheim E., Baghirov H., von Haartman E., Boe A., Aslund A.K., Morch Y., Davies Cde L. (2016). Cellular uptake and intracellular degradation of poly(alkyl cyanoacrylate) nanoparticles. J. Nanobiotechnol..

[5] Dobrovolskaia M.A., McNeil S.E. (2013). Understanding the correlation between in vitro and in vivo immunotoxicity tests for nanomedicines. J. Control. Release.

[6] Nel A., Xia T., Meng H., Wang X., Lin S., Ji Z., Zhang H. (2013). Nanomaterial toxicity testing in the 21st century: Use of a predictive toxicological approach and high-throughput screening. Acc. Chem. Res..

[7] Jones C.F., Grainger D.W. (2009). In vitro assessments of nanomaterial toxicity. Adv. Drug Deliv. Rev..

[8] Tekle C., van Deurs B., Sandvig K., Iversen T.G. (2008). Cellular trafficking of quantum dot-ligand bioconjugates and their induction of changes in normal routing of unconjugated ligands. Nano Lett..

[9] Grossman J.H., McNeil S.E. (2011). Preclinical Efficacy and Toxicity Testing of Engineered Nanomaterials. Nanomedicine-Basic and Clinical Applications in Diagnostics and Therapy.

[10] Pradines B., Lievin-Le Moal V., Vauthier C., Ponchel G., Loiseau P.M., Bouchemal K. (2015). Cell line-dependent cytotoxicity of poly(isobutylcyanoacrylate) nanoparticles coated with chitosan and thiolated chitosan: Insights from cultured human epithelial HeLa, Caco2/TC7 and HT-29/MTX cells. Int. J. Pharm..

[11] Kolter M., Ott M., Hauer C., Reimold I., Fricker G. (2015). Nanotoxicity of poly(n-butylcyano-acrylate) nanoparticles at the blood-brain barrier, in human whole blood and in vivo. J. Control. Release.

[12] Lherm C., Muller R.H., Puisieux F., Couvreur P. (1992). Alkylcyanoacrylate Drug Carriers. 2. Cytotoxicity of Cyanoacrylate Nanoparticles with Different Alkyl Chain-Length. Int. J. Pharm..

[13] Aslund A.K., Sulheim E., Snipstad S., von Haartman E., Baghirov H., Starr N., Kvale Lovmo M., Lelu S., Scurr D., Davies C.L. (2017). Quantification and Qualitative Effects of Different PEGylations on Poly(butyl cyanoacrylate) Nanoparticles. Mol. Pharm..

[14] NCI-NCL. https://ncl.cancer.gov/resources/assay-cascade-protocols.

[15] Damke H., Gossen M., Freundlieb S., Bujard H., Schmid S.L. (1995). Tightly regulated and inducible expression of dominant interfering dynamin mutant in stably transformed HeLa cells. Methods Enzymol..

[16] Lira M.C.B., Santos-Magalhaes N.S., Nicolas V., Marsaud V., Silva M.P.C., Ponchel G., Vauthier C. (2011). Cytotoxicity and cellular uptake of newly synthesized fucoidan-coated nanoparticles. Eur. J. Pharm. Biopharm..

[17] Kroll A., Dierker C., Rommel C., Hahn D., Wohlleben W., Schulze-Isfort C., Gobbert C., Voetz M., Hardinghaus F., Schnekenburger J. (2011). Cytotoxicity screening of 23 engineered nanomaterials using a test matrix of ten cell lines and three different assays. Part. Fibre Toxicol..

[18] Hanahan D., Weinberg R.A. (2011). Hallmarks of Cancer: The Next Generation. Cell.

[19] Yang Z.J., Chee C.E., Huang S., Sinicrope F.A. (2011). The role of autophagy in cancer: Therapeutic implications. Mol. Cancer Ther..

[20] Crist R.M., Grossman J.H., Patri A.K., Stern S.T., Dobrovolskaia M.A., Adiseshaiah P.P., Clogston J.D., McNeil S.E. (2013). Common pitfalls in nanotechnology: Lessons learned from NCI’s Nanotechnology Characterization Laboratory. Integr. Biol..

[21] Studer A.M., Limbach L.K., van Duc L., Krumeich F., Athanassiou E.K., Gerber L.C., Moch H., Stark W.J. (2010). Nanoparticle cytotoxicity depends on intracellular solubility: Comparison of stabilized copper metal and degradable copper oxide nanoparticles. Toxicol. Lett..

[22] Park M.V.D.Z., Neigh A.M., Vermeulen J.P., de la Fonteyne L.J.J., Verharen H.W., Briede J.J., van Loveren H., de Jong W.H. (2011). The effect of particle size on the cytotoxicity, inflammation, developmental toxicity and genotoxicity of silver nanoparticles. Biomaterials.

[23] Jiang W., Kim B.Y.S., Rutka J.T., Chan W.C.W. (2008). Nanoparticle-mediated cellular response is size-dependent. Nat. Nanotechnol..

[24] Chen H.T., Kim S.W., Li L., Wang S.Y., Park K., Cheng J.X. (2008). Release of hydrophobic molecules from polymer micelles into cell membranes revealed by Forster resonance energy transfer imaging. Proc. Natl. Acad. Sci. USA.

[25] Baghirov H., Melikishvili S., Morch Y., Sulheim E., Aslund A.K., Hianik T., de Lange Davies C. (2016). The effect of poly(ethylene glycol) coating and monomer type on poly(alkyl cyanoacrylate) nanoparticle interactions with lipid monolayers and cells. Colloids Surf. B Biointerfaces.

[26] Snipstad S., Hak S., Baghirov H., Sulheim E., Morch Y., Lelu S., von Haartman E., Back M., Nilsson K.P., Klymchenko A.S. (2016). Labeling nanoparticles: Dye leakage and altered cellular uptake. Cytom. A.

[27] Snipstad S., Westrom S., Morch Y., Afadzi M., Aslund A., de Lange Davies C. (2014). Contact-mediated intracellular delivery of hydrophobic drugs from polymeric nanoparticles. Cancer Nanotechnol..

[28] Korolchuk V.I., Saiki S., Lichtenberg M., Siddiqi F.H., Roberts E.A., Imarisio S., Jahreiss L., Sarkar S., Futter M., Menzies F.M. (2011). Lysosomal positioning coordinates cellular nutrient responses. Nat. Cell Biol..

[29] Kuempel E.D., Geraci C.L., Schulte P.A. (2012). Risk Assessment and Risk Management of Nanomaterials in the Workplace: Translating Research to Practice. Ann. Occup. Hyg..

[30] Leso V., Fontana L., Mauriello M.C., Iavicoli I. (2017). Occupational Risk Assessment of Engineered Nanomaterials: Limits, Challenges and Opportunities. Curr. Nanosci..

[31] Mørch Y., Hansen R., Berg S., Åslund A.K.O., Glomm W.R., Eggen S., Schmid R., Johnsen H., Kubowicz S., Snipstad S. (2015). Nanoparticle-Stabilized Microbubbles for Multimodal Imaging and Drug Delivery. Contrast Media Mol. Imaging.

[32] Klymchenko A.S., Roger E., Anton N., Anton H., Shulov I., Vermot J., Mely Y., Vandamme T.F. (2012). Highly lipophilic fluorescent dyes in nano-emulsions: Towards bright non-leaking nano-droplets. RSC Adv..

[33] Åslund A., Sigurdson C.J., Klingstedt T., Grathwohl S., Bolmont T., Dickstein D.L., Glimsdal E., Prokop S., Lindgren M., Konradsson P. (2009). Novel Pentameric Thiophene Derivatives for in Vitro and in Vivo Optical Imaging of a Plethora of Protein Aggregates in Cerebral Amyloidoses. ACS Chem. Biol..

