# Supplementary material for: Cytotoxicity of Poly(Alkyl Cyanoacrylate) Nanoparticles
Source: Int J Mol Sci. 2017 Nov 18;18(11):2454. doi: 10.3390/ijms18112454 (PMC5713421; doi:10.3390/ijms18112454)
Supplement: Supplementary file 1 [file ijms-18-02454-s001.pdf]

Cytotoxicity of Poly(Alkyl Cyanoacrylate) Nanoparticles

Einar Sulheim [1,2,*,†], Tore-Geir Iversen [3,4,†], Vu To [1], Geir Klinkenberg [1], Håvard Sletta [1], Ruth Schmid [1], Anne Rein Hatletveit [1], Ane Marit Wågbø [1], Anders Sundan [5], Tore Skotland [3,4], Kirsten Sandvig [3,4,6] and Ýrr Mørch [1]

Supplementary Material

Nanoparticle Synthesis

An oil-in-water emulsion was prepared by mixing a monomer oil phase with a water phase (0.1M HCl) containing two of the following PEG stabilizers: Brij® L23 (Polyoxyethylene lauryl ether, 5mM), Pluronic® F68 (polyoxyethylene-polyoxypropylene block copolymer, 2.5 mM), Kolliphor® HS15 (polyethylene glycol (15)-hydroxystearate, 5 mM), and Jeffamine® M-2070 (polyoxyethylene-polyoxypropylene-polyoxyethylene amine block copolymer, 68 mM, kindly provided by Huntsman Corporation, USA). The monomer phase contained either BCA, EBCA, or OCA, (kindly provided by Henkel Loctite, UK), as well as a neutral oil as co-stabilizer (Miglyol®810N, 2% *w*/*w* Cremer, Germany), a radical initiator (V65, Azobisdimetyl valeronitril, Wako, Germany, 0.9 wt%) and 0.2 wt% of either the fluorescent dyes pHTAM (12) (oligothiophene, a kind gift from Dr. Andreas Åslund, Norwegian University of Science and Technology) or NR668 (13). pHTAM is an oligothiophene and was a kind gift from Dr. Andreas Åslund, Norwegian University of Science and Technology. NR668 is modified Nile Red and was a kind gift from Dr. Andrey Klymchenko, University of Strasbourg. The chemical structures of the monomers were controlled using nuclear magnetic resonance (not shown). An oil-in-water nanoemulsion was achieved by sonification on an ice bath for 2 min (ultrasonifier, Branson, USA, 50% amplitude). Jeffamine® and Kolliphor® act both as PEG stabilizers and initiators as they contain a hydrophobic chain with a reactive amine (Jeffamine®) or hydroxyl (Kolliphor®) group which initiates polymerization at the droplet surface. In the cases where Jeffamine® were used, it was added right after sonication to avoid premature polymerization. Polymerization was carried out at ambient temperatures overnight. Potential unreacted monomer in the particle core was polymerized by increasing the temperature to 50 °C for 8 h, activating free radical polymerization by V65. The particles were rinsed by extensive dialysis to remove unbound stabilizers.

S1: Toxicity of Surfactants Used

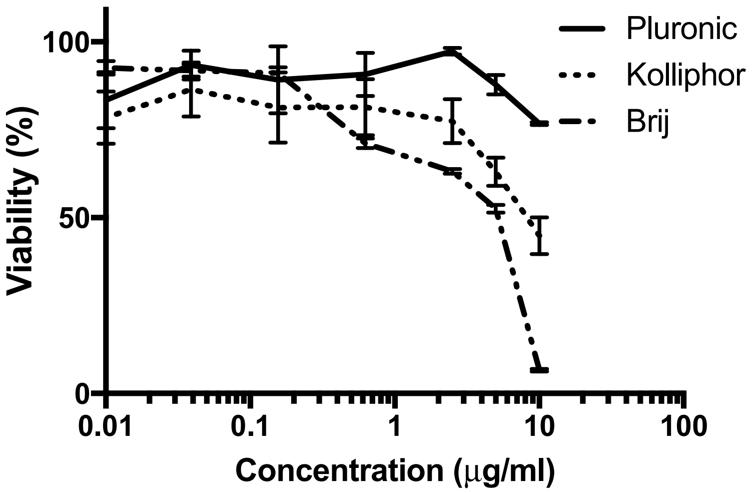

Figure S1. ATP content in HeLa cells after 24 h incubation with various surfactants used in the particles in this study, measured with the CellTiter-Glo® assay. N = 3 and error bars show standard deviation.

Cell Lines and Medium

Cell lines	Cell medium	Seeding density (cells/ml)
IMR-90	EMEM*	$5.4x10^4$
Hep G2	RPMI 1640**	$1x10^5$
RBE4	Ham's F-10***	$1x10^5$
DU-145	RPMI 1640**	$1x10^5$
A498	RPMI 1640**	$3.6x10^4$
SW-620	RPMI 1640**	$2.5x10^5$
HOP-92	RPMI 1640**	$5x10^4$
MDA-MB-468	RPMI 1640****	$3x10^5$
SF-295	RPMI 1640**	$5x10^4$
UACC-62	RPMI 1640**	$4.5x10^4$
OVCAR-3	RPMI 1640**	$8x10^4$
COLO 205	RPMI 1640**	$2x10^5$

*Supplemented with 10% FBS, 2 mM l-glutamine, non essential amino acids, 1 mM sodium pyruvate and 100 U/mL penicillin-streptomycin.

** Supplemented with 10% FBS, 2 mM l-glutamine, 10 mM HEPES and 100 U/mL Penicillin-Streptomycin

*** Supplemented with 10% FBS, 300 µg/ml G418 and 1 ng/ml basic fibroblast growth factor.

****Supplemented with 5% FBS, 2 mM l-glutamine, 10 mM HEPES and 100 U/mL Penicillin-Streptomycin

Table S1. Medium and seeding densities used for the 12 cell lines used for high-throughput cytotoxicity screening in 384-well trays.

Eagle's Minimum Essential Medium (EMEM) and Ham's F10 medium was bought from ThermoFisher, RPMI 1640 from Gibco. IMR-90 and Hep G2 were bought from ATCC/LGC standards. DU 145, A498, SW-620, HOP-92, MDA-MB-468, SF-295, UACC-62, OVCAR-3, and COLO 205 were all obtained from NCI-NCL and are part of the "NCI-60 Human Tumor Cell Lines Screen". RBE4 cells were a kind from Dr. Aschner, Vanderbilt University, Nashville, Tennessee. The HeLa cells (human cervical adenocarcinoma, Institut Curie Paris, France, 94% identical to ATCC HeLa cells) were grown in DMEM with 10% FBS; HeLa dynK44A cells (a kind gift from Sandra L. Schmid, UT Southwestern Medical Center, Dallas, Texas) were grown in DMEM with 10% FBS supplemented with geneticin (400 µg/mL), puromycin (200 µg/mL), and with or without tetracycline (1 µg/mL).

S2: Sterility and Immunology

Materials and Methods

Endotoxin content in PACA NP samples (2–200 μg/mL) was measured by applying Limulus Amebocyte Lysate (LAL) assay (Lonza Inc. Walkerwill, MD, USA, cat. No. 50-647U) according to the manufacturer's procedure.

Peripheral blood mononuclear cells (PBMC) were isolated from human A^+ buffycoats (The Bloodbank, St.Olav's University Hospital, Trondheim, Norway) with Lymphoprep as earlier described (Samstad, 2014). Monocytes were isolated by adherence to plastic (60 min AT 37 °C) in RPMI 1640 medium supplemented with 5% pooled human A^+ serum (The Bloodbank). Monocytes were incubated with controls (PBS as negative control and LPS and Zymosan as positive controls) and PIHCA NPs (2–20 μg/mL) for 6 h, before supernatants were harvested and analyzed for TNF- α by ELISA (R and D Systems Human TNF-α DuoSet ELISA, cat. no. DY 210).

Whole blood assays were performed essentially as described previously (Mollnes, 2002, Samstad, 2014). Briefly, blood was incubated with PEBCA NPs (up to 50 μg/mL) and controls (PBS as negative control and LPS and Zymosan as positive controls). Aliquots of blood were collected and analyzed for changes in cell surface marker (CD11b, 15 min), and cytokine production (Multiplex ELISA, 6 h).

Table S2. Stimulation of cytokine production in whole blood measured by ELISA 6 h after exposure to PEBCA NPs. Cytokine concentrations are given in pg/mL. *Value = value extrapolated beyond standard range; OOR> = out of range above; OOR< = out of range below; T0 = blank.

	PBS	Zymosan 10 µg/mL	PEBCA 50 µg/mL	PEBCA 20 µg/mL	PEBCA 10 µg/mL	LPS 0.1 µg/mL	T0
**IL-1beta**	4.74	167.90	7.47	10.97	9.32	371.50	*0.29
**IL-1ra**	872.54	2280.75	860.28	1696.90	1327.06	2623.51	86.15
**IL-2**	1.47	7.26	0.96	2.59	1.47	10.55	1.83
**IL-4**	2.67	5.14	2.39	3.02	2.81	6.82	0.67
**IL-5**	1.71	OOR<	OOR<	OOR<	OOR<	OOR<	OOR<
**IL-6**	2.34	2603.73	5.52	6.45	4.92	4964.48	*0.18
**IL-7**	1.94	3.16	6.71	4.36	5.54	4.36	OOR <
**IL-8**	630.64	4198.76	938.75	1465.26	1482.39	3490.54	2.71
**IL-9**	175.18	226.34	160.74	169.61	172.95	279.07	7.80
**IL-10**	9.17	14.58	7.11	8.13	7.11	16.82	*0.01
**IL-12 (p70)**	37.35	28.00	26.70	28.00	26.70	33.29	*1.07
**IL-13**	*1.68	*1.68	2.88	*1.93	*1.44	*1.68	OOR <
**IL-15**	21.11	57.86	12.70	25.83	35.00	64.72	OOR <
**IL-17**	825.40	1014.68	681.45	734.04	830.22	1149.41	40.19
**Eotaxin**	186.94	332.31	137.81	154.24	170.61	372.33	162.43
**Basic FGF**	77.63	99.52	107.11	85.95	95.08	116.56	34.54
**G-CSF**	16.99	88.47	25.18	27.91	27.91	155.06	25.18
**GM-CSF**	60.15	83.40	55.16	65.17	69.80	88.54	29.64
**IFN-gamma**	75.32	224.19	65.03	75.32	85.67	333.89	34.70
**IP-10**	4354.01	39,193.17	3124.27	4973.08	4259.57	53,195.94	2177.13
**MCP-1 (MCAF)**	176.26	282.20	77.85	94.09	152.53	182.42	33.62
**MIP-1alfa**	32.30	OOR>	117.39	135.23	126.00	OOR >	*1.08
**MIP-1beta**	3122.97	*27,010.70	7149.28	12,965.20	6312.92	OOR >	36.21
**PDGF-BB**	14,311.74	9515.01	3118.50	5778.25	6718.13	14,501.60	315.26
**RANTES**	17,782.56	17,018.60	6435.81	13,042.76	16,309.09	16,454.71	4276.46
**TNF-alfa**	41.50	502.04	34.85	44.87	48.27	499.80	15.87
**VEGF**	205.45	173.88	158.42	207.91	193.20	273.52	10.95

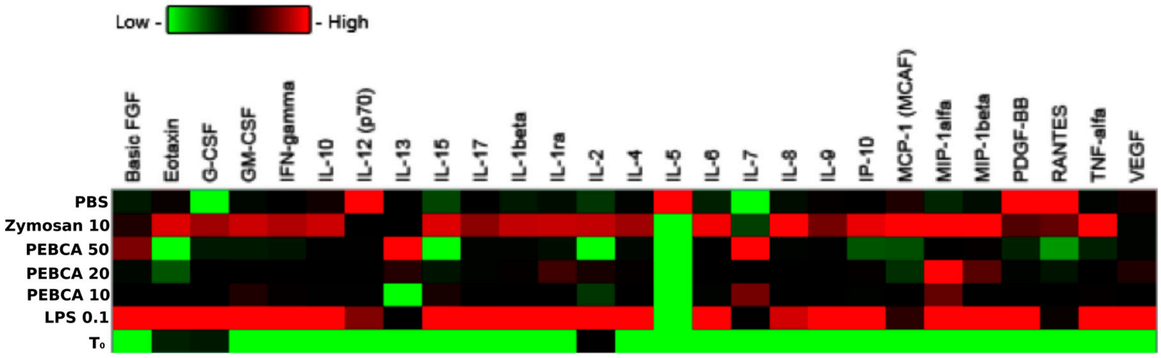

Figure S2. Heat map of cytokine production in whole blood when incubated with PEBCA NPs. Concentration of PIHCA NPs was 50, 20, and 10 µg/mL (designated as PEBCA 50, 20, and 10 in the heat map, respectively).

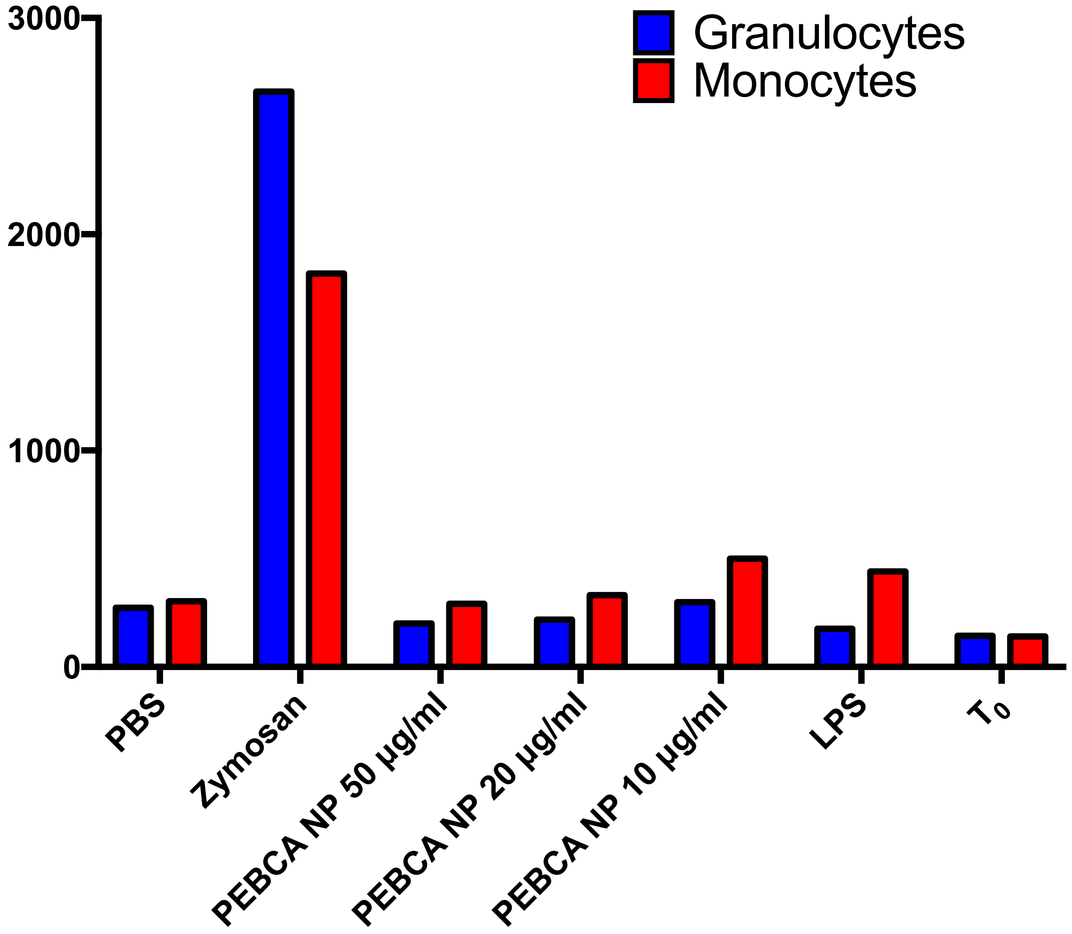

Figure S3. Measurements of CD11b in granulocytes and monocytes upon incubation with PEBCA NPs using a human whole blood assay.

References in Supplementary Material

MOLLNES, T. E., BREKKE, O. L., FUNG, M., FURE, H., CHRISTIANSEN, D., BERGSETH, G., VIDEM, V., LAPPEGARD, K. T., KOHL, J. & LAMBRIS, J. D. 2002. Essential role of the C5a receptor in E-coli-induced oxidative burst and phagocytosis revealed by a novel lepirudin-based human whole blood model of inflammation. *Blood,* 100**,** 1869-1877.

SAMSTAD, E. O., NIYONZIMA, N., NYMO, S., AUNE, M. H., RYAN, L., BAKKE, S. S., LAPPEGARD, K. T., BREKKE, O. L., LAMBRIS, J. D., DAMAS, J. K., LATZ, E., MOLLNES, T. E. & ESPEVIK, T. 2014. Cholesterol Crystals Induce Complement-Dependent Inflammasome Activation and Cytokine Release. *Journal of Immunology,* 192**,** 2837-2845.
